# Dynamics of Mitochondrial Transport in Axons

**DOI:** 10.3389/fncel.2016.00123

**Published:** 2016-05-13

**Authors:** Robert F. Niescier, Sang Kyu Kwak, Se Hun Joo, Karen T. Chang, Kyung-Tai Min

**Affiliations:** ^1^Department of Biological Sciences, School of Life Sciences, Ulsan National Institute of Science and TechnologyUlsan, South Korea; ^2^Department of Chemical Engineering, School of Energy and Chemical Engineering, Ulsan National Institute of Science and TechnologyUlsan, South Korea; ^3^Center for Multidimensional Carbon Materials, Institute for Basic ScienceUlsan, South Korea; ^4^Zilkha Neurogenetic Institute and Department of Cell and Neurobiology, University of Southern CaliforniaLos Angeles, CA, USA

**Keywords:** axonal transport, models, theoretical, mitochondrial transport, anterograde transport, retrograde transport

## Abstract

The polarized structure and long neurites of neurons pose a unique challenge for proper mitochondrial distribution. It is widely accepted that mitochondria move from the cell body to axon ends and vice versa; however, we have found that mitochondria originating from the axon ends moving in the retrograde direction never reach to the cell body, and only a limited number of mitochondria moving in the anterograde direction from the cell body arrive at the axon ends of mouse hippocampal neurons. Furthermore, we have derived a mathematical formula using the Fokker-Planck equation to characterize features of mitochondrial transport, and the equation could determine altered mitochondrial transport in axons overexpressing parkin. Our analysis will provide new insights into the dynamics of mitochondrial transport in axons of normal and unhealthy neurons.

## Introduction

Mitochondrial trafficking in axons is a widely documented phenomenon (Hollenbeck, 1996; Ligon and Steward, 2000; Chada and Hollenbeck, 2003; Miller and Sheetz, 2004; Chang and Reynolds, 2006; Miller and Sheetz, 2006; O'Toole et al., 2008; Saotome et al., 2008; Chang et al., 2011; Saxton and Hollenbeck, 2012), though the purpose behind bidirectional transport is unclear. A popular theory suggests that anterograde transport of mitochondria from the cell body to the synaptic terminals is essential for the delivery of these energy producing organelles to sites of high energy demand, whereas retrograde transport of mitochondria from the synaptic terminals to cell body is required for the removal of damaged mitochondria (Hollenbeck, 1996; Ligon and Steward, 2000; Chada and Hollenbeck, 2003; Miller and Sheetz, 2004; Chang and Reynolds, 2006; Saotome et al., 2008; Chang et al., 2011; Saxton and Hollenbeck, 2012). Consequently, defective mitochondrial transport and clearance can have deleterious effects on neuronal functions and have been associated with several neurological disorders (Schon and Przedborski, 2011). Despite this theory, however, tracking individual mitochondrial in the context of their origin (those originating distal or proximal to the soma) has not been examined, largely due to difficulties in monitoring mitochondria as they move pass each other in axons. Only a few studies to date have examined the bi-directional transport of mitochondria in the entire axon and over an extended time scale (O'Toole et al., 2008). Instead, most previous works on mitochondrial transport have monitored a short segment of axon less than 150 μm for 10 min (or less), which may not provide an accurate representation of anterograde and retrograde mitochondrial movement in axons. Previous studies have also reported a wide range of velocity averages for both anterograde and retrograde movement, likely due to different axonal areas monitored and how anterograde and retrograde movement was defined (MacAskill and Kittler, 2010).

Here, we have used a photo-switchable fluorescent protein to track the movement of mitochondria in the entire axon across an extended time frame, and developed a new assay to represent the velocity distribution of moving mitochondria. Furthermore, we have derived a mathematical equation to reliably characterize mitochondrial movement in axons.

## Results

### Velocity distribution of axonal mitochondria is determined by location

**Figure 1:**
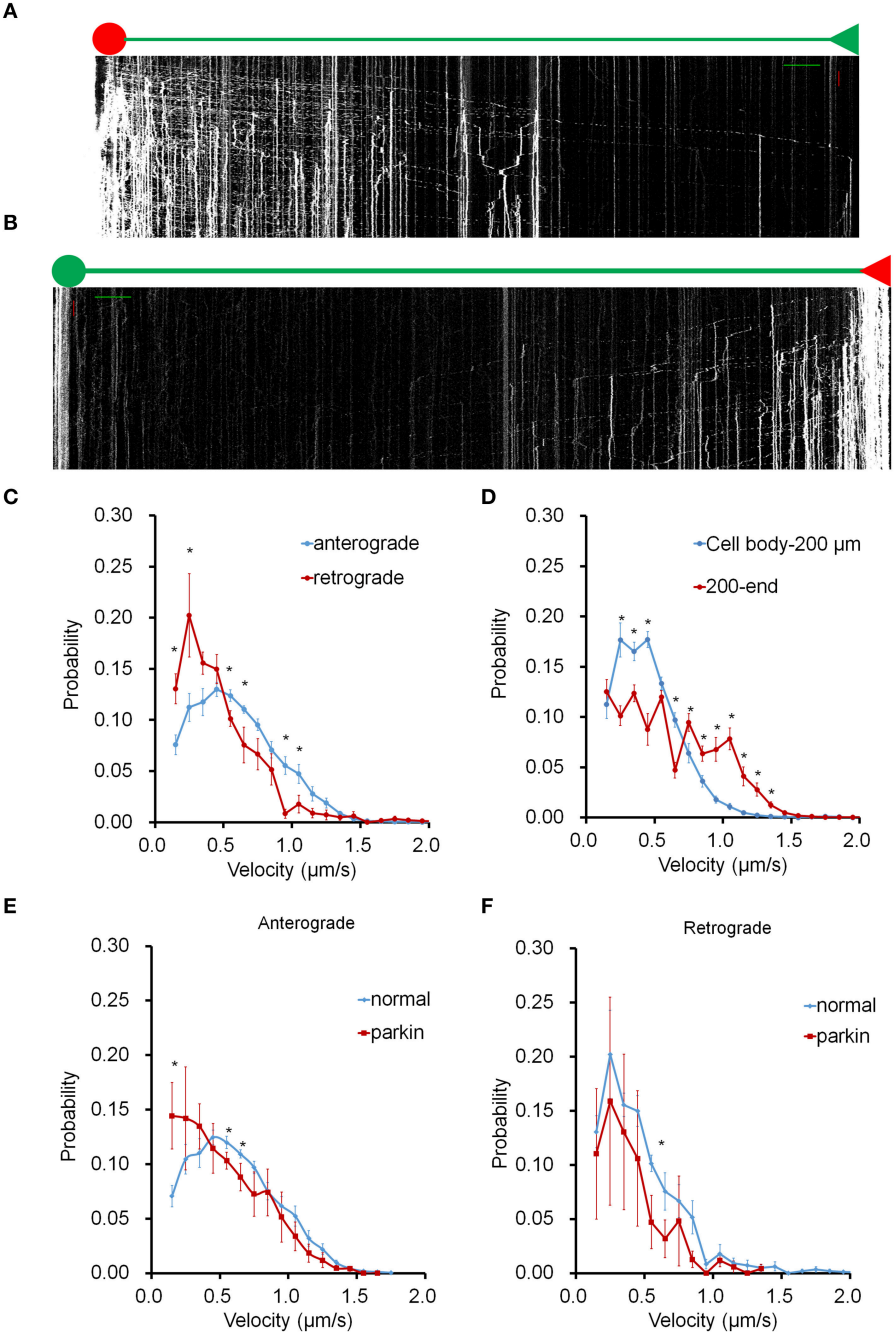
**Mitochondrial transport in the entire axon**. Mito-dendra2 was photoswitched at either the soma (anterograde) or an axonal endpoint (retrograde). **(A)** Kymograph for anterograde movement. Axon branch length: 1.064 mm. Scale bars: 50 μm for distance (green line), 15 min for time (red line). **(B)** Kymograph for retrograde movement. Axon branch length: 1.162 mm. Note that the dim red signal seen in the soma is due to the long emission tail of unconverted dendra2 and represent mitochondria already present in the soma prior to photo-activation. This signal is typically negligible; however, the cumulative brightness of hundreds of mitochondria in the soma can exhibit a red background. **(C)** Velocity distribution of anterograde and retrograde moving mitochondria. **(D)** Comparison of velocity distribution of mitochondria traveling within 200 μm of soma, and mitochondria traveling from 200 μm from the soma to the end. Anterograde forward movement proximal to the cell body was found to be slower than other areas of the axon. **(E,F)** Velocity distribution of mitochondrial movement in axon is compared between normal neuron and neuron overexpressing parkin. Anterograde movement is much slower in axon overexpressing parkin compared to that of normal axon **(E)**. Retrograde movement shows similar velocity distribution between normal and parkin overexpression **(F)**. *N* = 10 primary neurons for wild type anterograde, and 7 for wild type retrograde. *N* = 4 (anterograde) and 3 (retrograde) for neurons overexpressing parkin. ^*^P < 0.03. Values shown are mean ± s.e.m., and tested for statistical significance by student's *t*-test.

To investigate the bi-directional transport of mitochondria in axons, we targeted a photo-switchable fluorescent protein, dendra2 to the mitochondrial matrix (mito-dendra2) (Chudakov et al., 2007). Following photo-conversion, this approach enabled us to track individual mitochondria originating from specific regions over an entire axon for an extended time period. Anterograde mitochondrial movement was examined in mito-dendra2 transfected hippocampal neurons by irreversibly converting green fluorescent mito-dendra2 to red fluorescence in the soma (Figure 1A). We also converted mito-dendra2 in the axon area most distal to the soma and tracked red fluorescent mitochondria throughout the axon to investigate retrograde mitochondrial transport (Figure 1B, Supplementary Figure 1). We first measured the average velocity of mitochondria moving in the anterograde and retrograde direction for our imaging condition, and found that the values we obtained are within the published range (0.61 μm/s ± 0.003 for anterograde and 0.55 μm/s ± 0.020 for retrograde. ^*^P < 0.01; values represent mean ± S.E.M. *n* = 9 neurons for anterograde, *n* = 7 neurons for retrograde mitochondria). Next, we determined the velocity of axonal mitochondria by adopting a method that enables us to examine the motility of individual mitochondria in the entire axon. We calculated the velocity of all moving mitochondria from the slope of kymograph lines, which were drawn short enough to be well fitted to trajectories of moving mitochondrion in the corresponding interval. Figures 1C,D show the velocity distribution for anterograde and retrograde mitochondrial movement in axons (Supplementary Table 1). The most probable speed for anterogradely moving mitochondria (~0.5 μm/s; Figure 1C) is found to be two times faster than that of the retrograde movement (~0.25 μm/s). In addition, mitochondria proximal to the cell body (0–200 μm) showed a sharp drop-off of velocity probability compared to mitochondria traveling in an area farther away (200 μm to end; Figure 1D). These results suggest that the location within the axon can play a significant role in the velocity distribution of axonal mitochondria. It was reported that FLAG-parkin overexpression did not significantly change mitochondrial velocity in both anterograde and retrograde direction (Wang et al., 2011). To further test this result, we also expressed FLAG-parkin in neurons and analyzed mitochondrial transport. Our data, however, showed that the velocity distribution of anterograde movement is significantly altered in the axon overexpressing FLAG-parkin compared to that of the wild type, but retrograde movement is not affected (Figures 1E,F).

### Mathematical modeling of mitochondrial movement using the Fokker-Planck equation

Next, we derived a mathematical equation that describes this velocity distribution of mitochondrial movement in axons. First, we assumed that the velocity of moving mitochondria randomly varies in axons. In a mathematical sense, it becomes a stochastic quantity, which can be represented by the probability distribution. Hence, using the Fokker-Planck equation that can explain physical movement of particle in a medium in terms of drift and diffusion motions (Risken, 1996), we formulated the velocity distribution of a mitochondrion in axon. A general equation of the velocity distribution was derived and expressed as follows (see Materials and Methods for full derivation):

$$\begin{array}{l} W\left(v\right)=\;Av^{n}e^{\left(-Bv^{n-m+1}\right)} \end{array}$$

where *A* and *B* are constants, and *n* and *m*, which exhibited excellent agreement with experimental data. This equation provides a new tool to analyze mitochondrial velocity distribution in axons.

**Figure 2:**
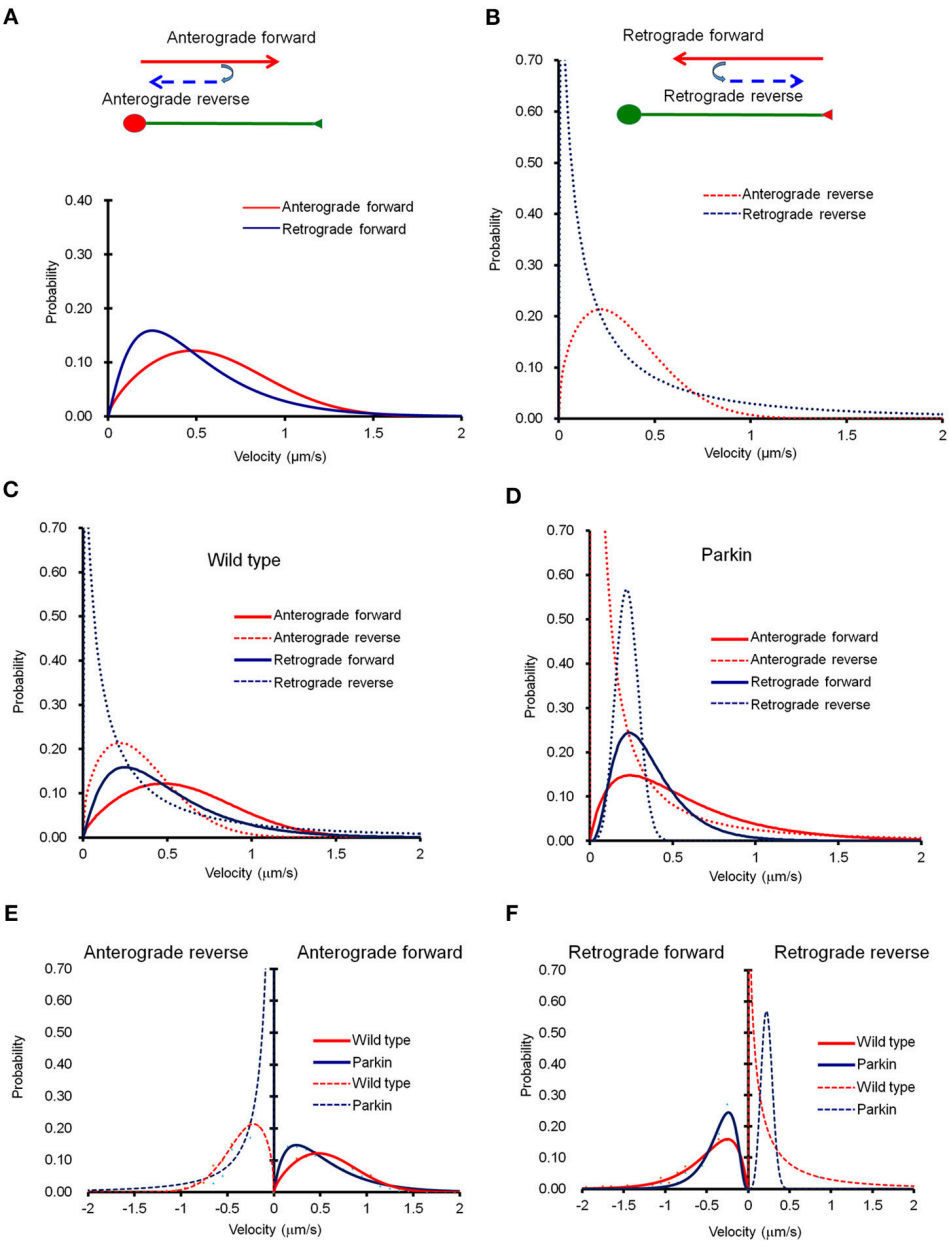
**The probability distribution function (PDF) derived from Fokker-Planck equation fits well into experimental data**. **(A)** Curve fit of anterograde movement. We defined anterograde forward as movement from the cell body toward the distal axon point, and anterograde reverse as movement that reverses in direction toward the cell body. Anterograde reverse (right) was consistent regardless of distance from the cell body, but was significantly slower than anterograde forward. **(B)** Retrograde forward was defined as movement from the axonal endpoint to the cell body, and retrograde reverse was opposite of the retrograde forward. A retrogradely forward moving mitochondrion is much faster than that of reverse. **(C,D)** Comparison mitochondrial transport in normal axons with that of axons overexpressing parkin. **(C–F)** Anterograde forward movement is much slower in axon overexpressing parkin compared to that of normal axon. Anterograde reverse direction is also slower due to parkin overexpression. Retrograde forward movement shows similar velocity distribution between normal and parkin overexpression. Interestingly, however, retrograde reverse movement in axon with parkin overexpression exhibits extremely narrow velocity distribution than that of normal axon. Anterograde reverse direction is also a little slower due to parkin overexpression. Retrograde forward movement shows similar velocity distribution between normal and parkin overexpression.

It is important to note that mitochondria also change the direction of their movement while traveling across the entire axon (Figure 2). When a mitochondrion reversed its direction, its velocity dropped significantly. Velocity distribution of anterograde reverse movement seems indistinguishable from that of retrograde forward movement, and could be mistaken as retrograde forward when the speed of mitochondria was tested in a short segment of axon for a brief period of time. Retrograde reverse movement is both rare and slow (Figure 2B). We also examined whether the mathematical equation can be used to characterize mitochondrial movement in axons with FLAG-parkin expression. The velocity distribution in the anterograde direction showed a difference within the velocity distribution curves (Figure 2C). However, retrograde movement appears to be largely unaffected (Figure 2D). Importantly, despite the shift in velocity distribution, our derived equation still fits the velocity distribution curve reliably, indicating the overall mitochondrial transport can be depicted by this mathematical equation (Figures 2C,D). Comparison of the velocity distribution of normal and Parkin overexpressing mitochondria shows that the velocity distribution is significantly altered in Parkin overexpressing mitochondria (Figures 2E,F). It is reported that Miro1, a regulator of mitochondrial movement can be degraded by Parkin (Wang et al., 2011; Birsa et al., 2014). Hence, it is plausible that overexpression of Parkin increases the rate of Miro degradation, and consequentially reduce overall mitochondrial velocity. Note that Figure 2F shows that Parkin overexpression causes an increase in retrograde reverse but a decrease in retrograde forward velocity. Since retrograde reverse is likely scored as an anterograde moving mitochondria in short-distance and short-duration imaging, this may explain why Wang et al. did not observe a change in anterograde velocity (Wang et al., 2011).

**Figure 3:**
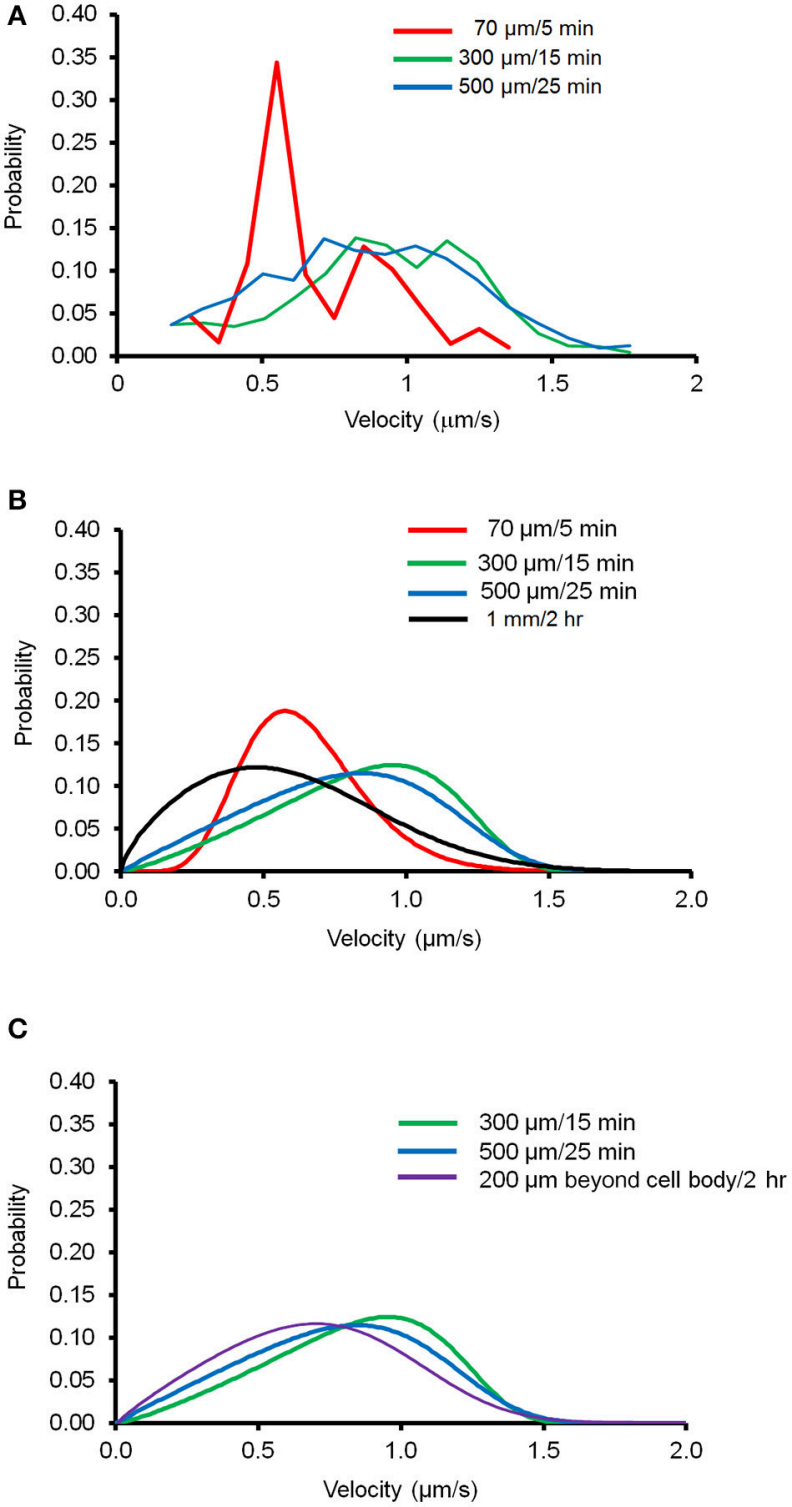
**Velocity distribution of mitochondrial transport in different experimental conditions**. Different length of axon segments and observation time were tested to determine the minimum experimental conditions necessary to generate velocity distribution of mitochondrial transport using the derivative of Fokker-Planck equation. **(A)** Velocity distribution of moving mitochondria plotted for experimental conditions as indicated. Data from eight different segments of axons were combined to calculate probability of velocity distributions. **(B)** Modeled velocity distribution plotted for each condition using the derivative of Fokker-Planck equation. **(C)** Comparison of modeled velocity distribution for data taken 200 μm beyond the cell body to the distal axonal area with the indicated condition.

Next, we tested the applicability of this newly derived mathematical equation by determining the minimum amount of imaging data necessary to reliably depict mitochondrial movement. We generated the velocity distribution of anterograde forward movement with three different lengths of axon segments and different period of time: 70 μm/5 min, 300 μm/15 min, and 500 μm/25 min. Eight different regions of existing kymographs of an axon were randomly chosen, which were used to prepare the velocity distribution for each case. Figure 3A shows the experimental data obtained for velocity distribution and Figure 3B shows the modeled velocity distribution using the derived Fokker-Planck equation. None of these conditions matched the distribution curve obtained for imaging the entire axon for an extended time frame. However, as mitochondria within 200 μm of cell body show altered mitochondrial movement (Figure 1D), we compared the modeled velocity distribution for axon 200 μm beyond the cell body. Figure 3C shows that both 300 μm /15 min and 500 μm /25 min closely matches the modeled distribution obtained for axon segment 200 μm from the cell body to the terminal. Together, these results suggest that the velocity distribution of mitochondrial transport for region 200 μm beyond the cell body may be accurately represented using a minimum 300 μm/15 min experimental condition and our newly derived equation.

### Mito-dendra2 as a marker for whole-axon mitochondrial dynamics

**Figure 4:**
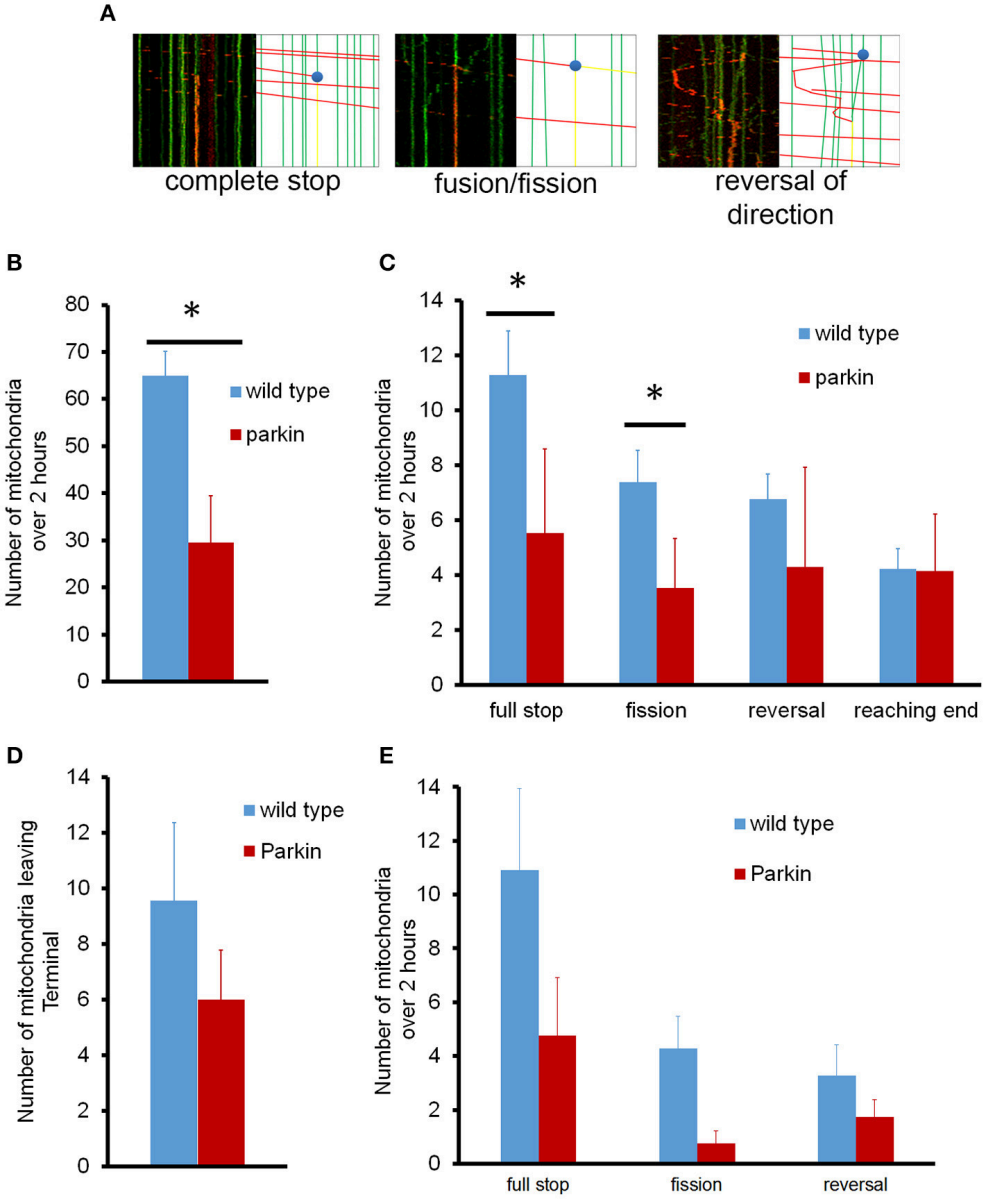
**Representative kymographs for mitochondrial events in normal neurons and neurons overexpressing parkin**. **(A)** Tagged mitochondria were tracked for dynamics events as they traveled either anterograde or retrograde through the axon over 2 h (i) full stop of mitochondria. (ii) fusion and fission. (iii) reversal of movement. **(B)** Quantitative analysis of anterograde mitochondrial transport in axons. **(C)** Dynamics of anterograde mitochondrial events in axons. For the fusion/fission event, a red moving mitochondrion is split into 1 stationary mitochondrion and 1 that continued its movement. **(D)** Number of mitochondria leaving the axonal endpoint over 2 h. **(E)** Dynamics of retrograde mitochondrial transport in axons. The number and destiny of mitochondria moving in the anterograde or retrograde direction were normalized to the length of the axon. *N* = 10 primary neurons for wild type anterograde, and 11 for wild type retrograde. *N* = 4 (anterograde) and 3 (retrograde) for neurons overexpressing parkin. ^*^P < 0.01.

We further analyzed the mitochondrial dynamics in the whole axon; mitochondria underwent fusion and fission, pausing, and reversal in while traveling along the axon (Figure 4A). First, we examined if mitochondria left the cell body indeed reach to the endpoint of the axon. The total number of mitochondria leaving the cell body during 2 h of imaging averaged 65 ± 5 mitochondria/axon, which were distributed to different axon branches (Figure 4B). We also examined the number of dynamic events that occurred within the axon (Figure 4C). Since hippocampal axons branch extensively and showed a total axonal length variation between 2 and 5 mm (about 3.2 mm of axon branches on average), we took all branches into consideration when characterizing mitochondria that leave the cell body. On average, about 4 mitochondria/mm of axon arrived at the axon endpoint, and 4 mitochondria/mm stopped completely without changing direction. Approximately 7 mitochondria/mm reversed in direction but ultimately paused, and 7 mitochondria/mm fused to stationary mitochondria and split but stopped later. Fusion of mitochondria can be readily observed as exchange of unconverted (green) and converted (red) mito-dendra2 signal. We noticed that for these mitochondria that fused and split has a tendency to move in the initial direction before pausing. Other mitochondria did not reach a destination or undergo another event, as we were not able to observe its fate within the time allotted. It is interesting to note that the majority of mitochondria that left the cell body underwent different fates rather than arriving at the furthest distal point. FLAG-Parkin overexpression, however, significantly reduced the number of mitochondria leaving the cell body (30 ± 10 mitochondria; Figure 4B), as well as the number of stopping and dividing mitochondria (Figure 4C), although the number of mitochondria reaching the end was not reduced.

Surprisingly, we found that none of the retrogradely moving mitochondria originating from the distal axonal area reached the cell body during 2 h of imaging period, which is incompatible to the model that retrogradely moving mitochondria arrive at the cell body for processing and repair (Maday et al., 2012). To verify if the 2 h observation time period is a limiting factor, we examined the retrograde movement of mitochondria for up to 16 h. Mitochondria that left the distal axonal area still did not reach the cell body following an extended period of imaging, confirming that retrogradely moving mitochondria indeed do not arrive to the cell body. Our data showed an average of 10 mitochondria left the terminal during 2 h. However, all of them stopped in the middle of axon (Figure 4D). Fusion/fission and reversal in direction were also observed (Figure 4E). Interestingly, mitochondria that stopped in the middle of axon always fused to an existing stationary mitochondrion and the number of mitochondria leaving the distal axonal area exceeds the number of mitochondria arriving there. These results suggest that axonal mitochondria may undergo dynamic mitochondrial biogenesis or fission.

### Analysis of stationary distribution in axons

**Figure 5:**
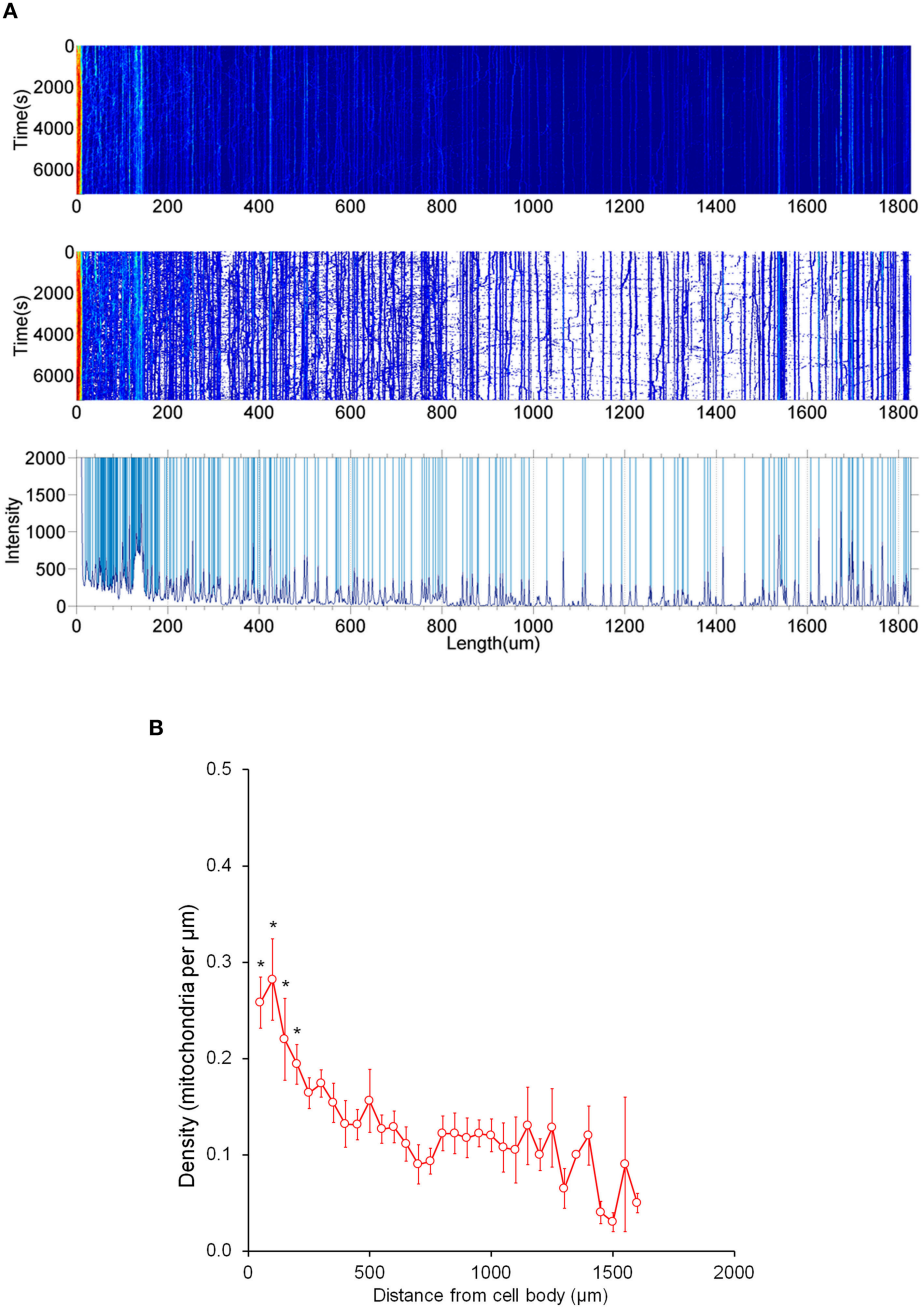
**Distribution of stationary mitochondria in the entire axon**. **(A)** Representative kymograph demonstrating analysis of stationary mitochondria. A typical kymograph (top) was thresholded to eliminate background (middle). Peaks that appeared constant over the course of 2 h were quantified as stationary (bottom). **(B)** Density of mitochondria throughout axon. Axonal lengths of 50 μm were binned, and the frequency of mitochondria in reference to distance from the cell body was plotted. Value = mean ± S.D. ^*^P < 0.02 compared to density at 750 μm. 750 μm was selected for statistical comparison because it is roughly the middle of the average axon length monitored. A longest axon in a neuron was analyzed. *N* = the longest branch from 10 primary neurons.

It is important to note that most mitochondria in the axon are not motile, but stationary; we therefore investigated the distribution pattern of stationary mitochondria in the entire primary axon. First, we filtered out fluorescent background and set up position peaks for stationary mitochondria (Figure 5A), and counted the number of stationary mitochondria over 50 μm. Our data showed that there is a dense distribution of stationary mitochondria in the axon segment proximal to the cell body (200 μm from the cell body), while the rest of axonal region contains less stationary mitochondria that are relatively uniform in density (Figure 5B). This higher density correlates with our velocity distribution analysis (Figure 1), which shows that mitochondria move more slowly closer to the cell body, and suggests that this change in velocity is caused through the physical obstructions caused by other mitochondria.

## Discussion

One key parameter used to characterize mitochondrial transport in the axon has been the speed of mitochondrial movement. Almost all previous studies used the average speed of individual moving mitochondria over the period of imaging, which varied from 0.1 to 1.4 μm/s (Morris and Hollenbeck, 1993; Miller and Sheetz, 2004; Jimenez-Mateos et al., 2006; Chen et al., 2007; Mironov, 2007; Misgeld et al., 2007; Kang et al., 2008; Wang and Schwarz, 2009; MacAskill and Kittler, 2010; Chang et al., 2011). Furthermore, several studies have previously reported that anterograde velocity is faster than that of retrograde movement, while others found opposite results, or no difference in the speed between anterograde and retrograde movement. By using photo-switchable fluorescent mitochondria, our studies provide a comprehensive analysis of mitochondrial transport in the axon. We have demonstrated that anterogradely moving mitochondria are faster than retrogradely moving mitochondria, and the velocity of anterograde movement varies along the region of the axon traveled. Mitochondria proximal to the cell body (0–200 μm) showed a sharp drop-off of velocity probability compared to mitochondria traveling in an area farther away (200 μm to end). These results suggest that the location within the axon can play a significant role in the velocity distribution of axonal mitochondria.

A previous report discussed the properties of retrogradely moving mitochondria, including the suggestion that damaged mitochondria move retrogradely toward the cell body for clearance (Miller and Sheetz, 2004), while others reported that the direction of mitochondria movement is not related to mitochondrial membrane potential (Gerencser and Nicholls, 2008; Verburg and Hollenbeck, 2008). Several reports also showed that damaged mitochondria could be removed by mitophagy in the distal part of the axon (Wang et al., 2011; Liu et al., 2012; Ashrafi et al., 2014), suggesting that mitochondria do not need to return to the cell body for targeted degradation of damaged mitochondria. Consistent with these results, we have found that anterograde moving mitochondria that left from the cell body arrive at distal axonal area, while retrograde mitochondria do not reach to cell body.

Using the probability distribution function (PDF) derived from Fokker-Planck equation, we have characterized mitochondrial transport in axon, and parameters of the PDF were obtained by fitting velocity distribution data using the least square fitting method (see Materials and Methods). Furthermore, mitochondrial transport in axons overexpressing FLAG-parkin showed significantly different patterns than those of wild type in the velocity range of moving mitochondria. The decrease in anterograde velocity may be due to the targeting of Miro1, a regulator of mitochondrial movement, by Parkin (Wang et al., 2011), as well as changes in motor activity or function. Together, the differences in the velocity distribution of mitochondrial transport provide new tools to distinguish and characterize normal and abnormal mitochondrial transport in healthy or diseased neurons. Our mathematical formula provides characterization of the velocity distribution of mitochondrial transport in axons. Proper integration of this formula to future studies will provide new insights into mitochondrial behavior in neurons from different neurological disorders such as Parkinson's, Alzheimer's, Charcot-Marie-Tooth diseases, Schizophrenia, as well as Down syndrome (Mattson et al., 2008; Du et al., 2010; Rintoul and Reynolds, 2010; Schon and Przedborski, 2011), in which altered mitochondrial transport in axons has been implicated.

## Materials and methods

### Animals

Animals were used in accordance with protocols approved by the Animal Care and Use Committees of UNIST. C57BL/6 mouse strain was purchased from Hyochang Science (Korea).

### Primary hippocampal cell culture

For primary cell culture, pregnant wild type mice were euthanized with CO_2_ 18 days past impregnation (E18). Embryos were collected, placed in ice-cold HBSS pH 7.2 (Invitrogen) and hippocampi were dissected from embryos. Extracted hippocampi were dissociated at 37°C in trypsin (Invitrogen) for 15 min, washed in HBSS once, trituration media (DMEM, 1% Penicillin/Streptavidin, 10% horse serum) twice, then plated on glass bottom dishes. After 3 h, media was replaced by maintenance media (Neurobasal media, B27, Glutamax) and cultured in 37°C, 5% CO_2_ incubator. Neurons were transfected with mito-dendra2 and/or FLAG-Parkin using Lipofectamine 2000 (Invitrogen) at DIV 4, and were imaged at DIV 13–17.

### Confocal microscopy

Cells were imaged using a Zeiss LSM 780 confocal microscope, kept at 37°C with an XL incubator for the duration of imaging. Prior to imaging, neurobasal media was replaced with Tyrode's Buffer (135.0 mM NaCl, 5.0 mM KCl, 1.8 mM CaCl_2_, 1.0 mM MgCl_2_, 10.0 mM HEPES, 5.5 mM glucose, pH 7.3). Axons were identified from dendrites by their longer lengths and smooth morphology. Lengths of 250 μm or more were used for analysis. After target cell was identified, axons were framed so that the majority of the axon could be imaged within two stitched frames and a three level z-stack. Mito-dendra-2 was photo-switched using a 405 nm laser at either the soma or the axon end. Cells were then imaged every 15 s for 2 h at 12 bits depth. For long-term imaging (16 h), cells were cultured in neurobasal media, kept at 37°C with 5% CO_2_ using a stage top incubator. Time lapses were stitched and projected using Zeiss ZEN 2011 software. For kymograph generation, axons were traced through a time projection by hand by following mitochondrial movement over time with a five pixel wide line. The ImageJ straighten function was used, and the straightened time lapse was binned maximally to condense the y-dimension to one pixel. The montage function was used to produce kymographs. Following axon tracing, axon length and intensities was converted to numerical values using the ImageJ StackReg plugin for further Matlab analysis.

### Parkin cloning

Parkin was amplified from total brain RNA using Superscript III one step RT-PCR system (Invitrogen) into the sigma pFLAG-CMV-6B vector. We used the primer sequences: 5′-GAGTCAAGCTTGATGATAGTGTTTGTCAGGTTCAAT-3′ and 5′-CGATCGAATTCCTACACGTCAAACCAGTGATCTCCA-3′.

### Analysis of mitochondrial motility

Velocity distribution of moving mitochondria was analyzed by measuring the velocity of a mitochondrion as it travels through the axon. First, straight lines are drawn on kymographs. All visible branch points were analyzed for mitochondrial velocity and destination. The length of each line is short enough for that to be well fitted in corresponding interval. Next, length and angle of each line are measured. Velocity of each mitochondrion was then calculated from the slope of line with equation below.

(1.1) $$\begin{array}{l} \text{Velocity }=\;\left(\cot\left(-\text{Angle}\right)\frac{pixel}{pixel}\right)\times \left(\frac{\left(\frac{\text{branch length}}{\text{kymograph width}}\right)\frac{um}{pixel}}{\left(\frac{\text{time lapse}}{\text{kymograph height}}\right)\frac{s}{pixel}}\right) \end{array}$$

Traveling time of a mitochondrion is calculated for velocity distribution with the following equation:

(1.2) $$\begin{array}{l} \text{Traveling Time }=\text{ abs}\left(\text{Length}\times \sin\left(\text{Angle}\right)\frac{pixel}{pixel}\right)\times \\ \;\left(\left(\frac{\text{time lapse}}{\text{kymograph height}}\right)\frac{s}{pixel}\right) \end{array}$$

Next, binning of all velocity data was done with a bin size of 0.1 μm/s followed by normalization of data. Each velocity data was weighted with traveling time of a mitochondrion with that velocity. Note that the velocity from 0 μm/s to 0.1 μm/s was treated as “stop.” The velocities of forward movement and reverse movement were separately normalized such that anterograde forward moving mitochondria were not considered when calculating anterograde reverse movement from the same cell.

### Derivation of Fokker-Planck equation

The velocity of moving mitochondria randomly varies in different axonal environment. In a mathematical sense, it becomes a stochastic quantity, which can be represented by the probability distribution (or probability density due to continuity of the velocity). We defined a distribution density W(v,t), where v is velocity and t is time. Then the equation of motion of W(v,t) can be expressed as follows,

(2.1) $$\begin{array}{l} \frac{\partial W}{\partial t}=-\frac{\partial }{\partial v}\left(fW\right)+\frac{\partial^{2}}{\partial v^{2}}\left(gW\right) \end{array}$$

where *f* and *g* are functions of velocity. The Equation (2.1) is known to be the Fokker-Planck equation (Risken, 1996). Note *W* is used instead W(v,t) hereafter. As *t*
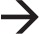
∞, *W* becomes stationary within the boundary condition of *v* as [0,v_max_], where the probability current is assumed to be zero. Then, one can extract the following expression from Equation (2.1),

(2.2) $$\begin{array}{l} \frac{\partial gW}{\partial v}-fW=0 \end{array}$$

We can rearrange Equation (2.2) as follow,

(2.3) $$\begin{array}{l} g\frac{\partial W}{\partial v}-\left(f-\frac{\partial g}{\partial v}\right)W=0 \end{array}$$

If we solve Equation (2.3) in terms of *W*,

(2.4) $$\begin{array}{l} W=\frac{1}{g}\exp\left(\int \frac{f}{g}dv\right) \end{array}$$

where *A* is constant. As *v*
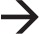
0, *W*
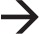
0. Thus, *g* (diffusion coefficient) must have an inverse function of *v*. The simplest form that we applied is *g* = *av*^−*n*^, where *a* and *n* are constants. Similarly, as *v*
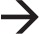
∞, *W*
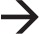
0. Thus, *f* (drift coefficient) must have a negative function of *v* since it is in the integral exponent. The simplest form that we applied is *f* = *bv*^−*m*^, where *b* and *m* are constants. Note the negative sign of *m* is chosen to compromise the inverse *g*. To this end, the final form of *W* is derived as follows,

(2.5) $$\begin{array}{l} W=Av^{n}\exp\left(-Bv^{n-m+1}\right)\left(A\;=\;\frac{C}{a},B=-\frac{b}{a\left(n-m+1\right)}\right) \end{array}$$

where *C* is integration constant. We investigated the effect on the velocity distribution by modifying *A, B, n*, and *m* in the normalized velocity distribution, where *A* is the function of *B, n*, and *m*. The equation is expressed as follows,

(2.6) $$\begin{array}{r} W\left(normalized\right)=\frac{W}{\overset{\infty }{\underset{0}{\int }}Wdv}=\frac{k}{B^{-\frac{1}{k}-\frac{n}{k}}\Gamma \left(\frac{1+n}{k}\right)}v^{n}e^{-Bx^{k}}\\ \left(v>0,n>0,B>0,k>0,k=n-m+1\right) \end{array}$$

When *B* increases, the velocity distribution becomes sharp and the peaks move to the left (*v*
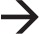
0). When *n* increases, the velocity distribution becomes broad and the peak moves to the right (*v*
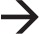
∞). Since *m* is in *k*, we consider varying *k* from *k*_min_ = 0.1 to *k*_max_ = 2 with an interval of 0.1 (i.e., dk) while fixing *B* (i.e., 1 and 5) and *n* (i.e., 0.1 to 10). If the velocity distribution is weighted toward *v* = 0, the increase of *m* makes the velocity distribution sharp and move to the left until *m* reaches to a certain value. Further increase of *m* makes the velocity distribution broad. If the velocity distribution is weighted far from *v* = 0, the increase of *m* makes the peak move to the right and the broad distribution.

### Stationary mitochondria analysis

Stationary mitochondria were analyzed using MATLAB. Intensity values smaller than 100 were made to 0 in order to filter out background. The intensity (*z*-value, color) along the axon was averaged over time. After that, local maxima were found by comparing peaks with their nearest neighboring values. The position of maximum peaks whose value is larger than 150 is identified as location of stationary mitochondria. Distribution of stationary mitochondria along the axon was obtained by counting the number of stationary mitochondria, which are found from the above method, in bins with interval size of 50 μm.

## Author contributions

RN performed all experiments, analyzed data and contributed to writing. SK and SJ formulated the mathematical equation, analyzed data and contributed to writing. KC provided a material and contributed to discussion and writing. KM conceived idea and wrote the manuscript.

## Funding

This work was supported by Samsung Science and Technology Foundation (SSTF-BA1301003). We declare no competing financial interests.

### Conflict of interest statement

The authors declare that the research was conducted in the absence of any commercial or financial relationships that could be construed as a potential conflict of interest.
