# Supplementary material for: Dynamics of Mitochondrial Transport in Axons
Source: Front Cell Neurosci. 2016 May 13;10:123. doi: 10.3389/fncel.2016.00123 (PMC4865487; doi:10.3389/fncel.2016.00123)
Supplement: Supplementary file 2 [file Presentation1.PDF]

Dynamics of mitochondrial transport in axons

Robert F. Niescier[*,1], Sang Kyu Kwak[*,2], Sehun Joo[2], Karen T. Chang[3], & Kyung-Tai Min[1]

[1]Department of Biological Sciences, School of Life Sciences, Ulsan National Institute of Science and Technology, Ulsan, Korea. [2]Department of Chemical Engineering, School of Energy and Chemical Engineering, Ulsan National Institute of Science and Technology, Ulsan, Korea. [3]Zilkha Neurogenetic Institute and Department of Cell and Neurobiology, University of Southern California, Los Angeles, CA, USA.

* These authors contributed equally to this work.

Correspondence and requests for materials should be addressed to K-T. M (ktaimin @unist.ac.kr)

Methods

Analysis of mitochondrial motility

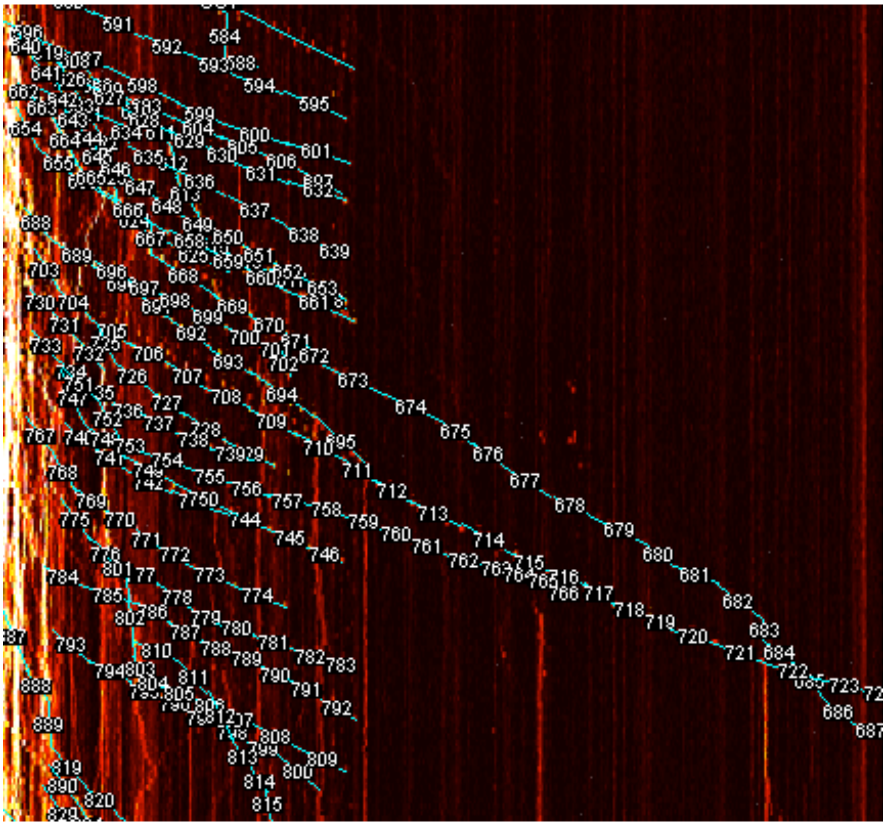

Velocity distribution of moving mitochondria was analyzed by measuring the velocity of a mitochondrion as it travels through the axon. At first, straight lines are drawn on the kymograph along the trajectories of moving mitochondria using ImageJ. The length of each line is short enough for that to be well fitted in corresponding interval. After that, length and angle of each line are measured in pixels.

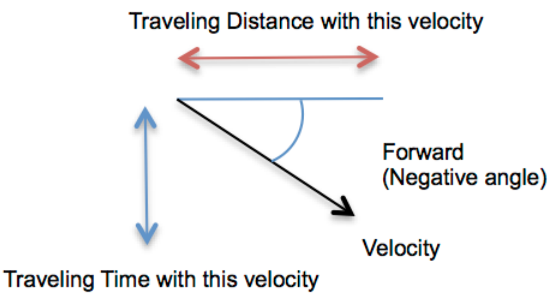

Velocity of each mitochondrion was then calculated from the slope of line with equation below.

$$\text{Velocity} = \left(\cot(-\text{Angle})\,\frac{pixel}{pixel}\right) \times \left(\frac{\left(\frac{\text{branch length}}{\text{kymograph width}}\right)\frac{um}{pixel}}{\left(\frac{\text{time lapse}}{\text{kymograph height}}\right)\frac{s}{pixel}}\right) \qquad (1.1)$$

Also, traveling time of a mitochondrion with each velocity is calculated for velocity distribution with equation (1.2).

$$\text{Traveling Time} = \text{abs}\left(\text{Length} \times \sin(\text{Angle})\,\frac{pixel}{pixel}\right) \times \left(\left(\frac{\text{time lapse}}{\text{kymograph height}}\right)\frac{s}{pixel}\right) \quad (1.2)$$

	Velocity	Time
Line 1	v_1	t_1
Line 2	v_2	t_2
Line 3	v_3	t_3
Line 4	v_4	t_4
Line 5	v_5	t_5
:	:	:
:	:	:
Line n	v_n	t_n

➡

Velocity	Time
$0.1 \le v < 0.2$	$\sum_{0.1 \le v < 0.2} t_v$
$0.2 \le v < 0.3$	$\sum_{0.2 \le v < 0.3} t_v$
$0.3 \le v < 0.4$	$\sum_{0.3 \le v < 0.4} t_v$
$0.4 \le v < 0.5$	$\sum_{0.4 \le v < 0.5} t_v$
$0.5 \le v < 0.6$	$\sum_{0.5 \le v < 0.6} t_v$
$0.6 \le v < 0.7$	$\sum_{0.6 \le v < 0.7} t_v$
:	:
:	:

➡

Velocity	Time
$0.1 \le v < 0.2$	$\frac{\sum_{0.1 \le v < 0.2} t_v}{t_{total}}$
$0.2 \le v < 0.3$	$\frac{\sum_{0.2 \le v < 0.3} t_v}{t_{total}}$
$0.3 \le v < 0.4$	$\frac{\sum_{0.3 \le v < 0.4} t_v}{t_{total}}$
$0.4 \le v < 0.5$	$\frac{\sum_{0.4 \le v < 0.5} t_v}{t_{total}}$
$0.5 \le v < 0.6$	$\frac{\sum_{0.5 \le v < 0.6} t_v}{t_{total}}$
$0.6 \le v < 0.7$	$\frac{\sum_{0.6 \le v < 0.7} t_v}{t_{total}}$
:	:
:	:

Afterward, binning of all velocity data was done with bin size of 0.1 μm /second followed by normalization of data. Each velocity data was weighted with traveling time of a mitochondrion with that velocity. Note that the velocity from 0 μm /second to 0.1 μm/second was treated as 'stop', thus not included in velocity distribution of moving mitochondria. And the velocities of forward movement and reverse movement were separately normalized.

2. Derivation of Fokker-Planck Equation

Normal axon

Anterograde movement

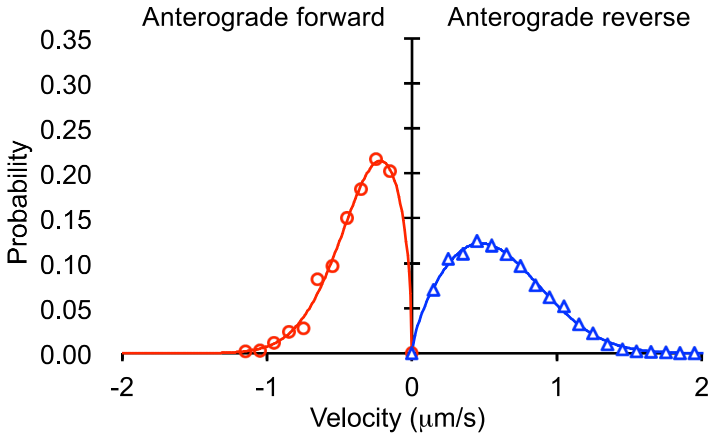

Retrograde movement

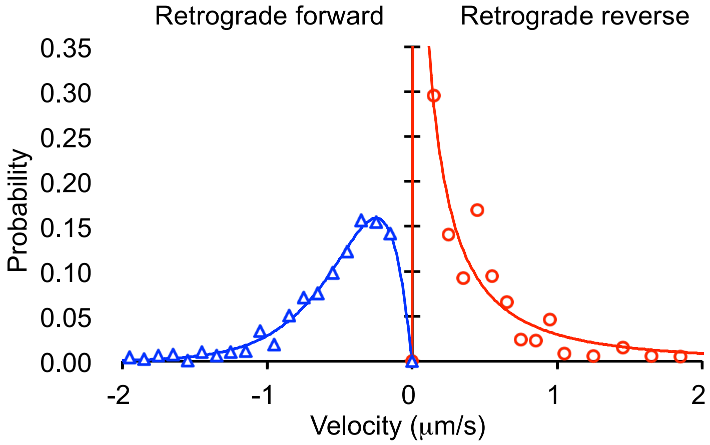

	A	B	n	m
Anterograde forward	0.268	1.612	0.671	-0.645
Anterograde reverse	0.551	4.284	0.460	-0.419
Retrograde forward	2.793	4.584	1.167	1.228
Retrograde reverse	15975.079	13.191	0.928	1.737

Axon overexpressing parkin

Anterograde movement

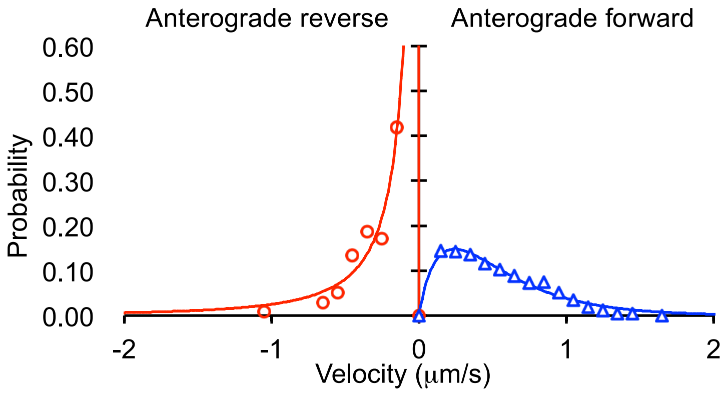

Retrograde movement

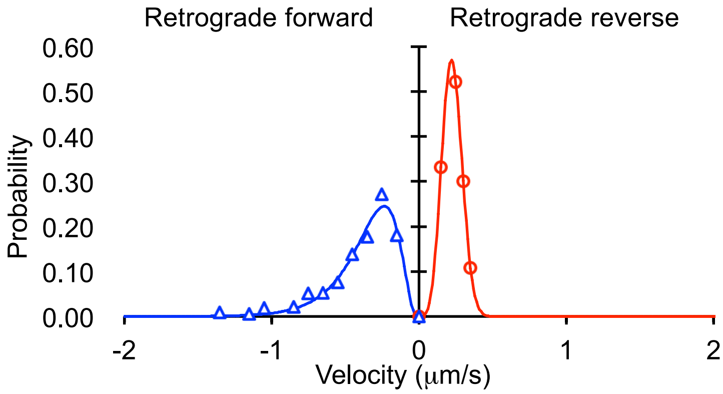

	A	B	n	m
Anterograde forward	1.744	3.819	0.964	1.087
Anterograde reverse	271159.278	16.192	0.783	1.621
Retrograde forward	163.715	10.244	2.551	2.663
Retrograde reverse	706.521	77.217	3.765	2.128

Supplementary Figures

Supplementary Figure 1: Ratio Red/Green image of retrograde movement kymograph from Figure 1B*.* The ratiometric image emphasizes that there is little spontaneous photo-activation from the imaging conditions used.
