# Supplementary figures and images for: Dynamics of Mitochondrial Transport in Axons
Source: Front Cell Neurosci. 2016 May 13;10:123. doi: 10.3389/fncel.2016.00123 (PMC4865487; doi:10.3389/fncel.2016.00123)

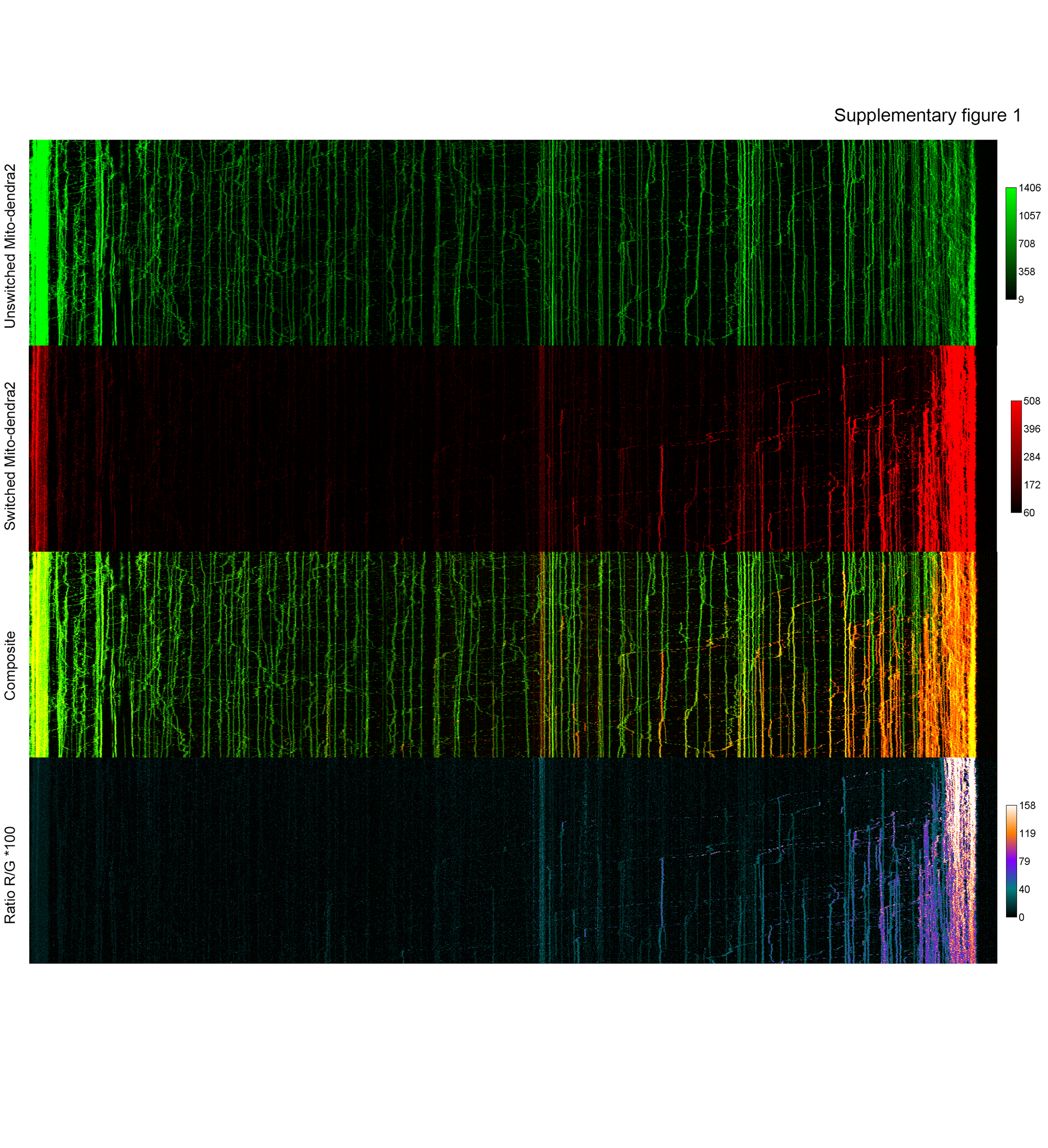

Supplement: Supplementary file 3 [file Image1.TIF]
